# From art to metrics: a closed-loop framework for quantifying dose, manipulation, and feedback

**DOI:** 10.1186/s13020-026-01492-z

**Published:** 2026-09-22

**Authors:** Wen-Chao Tang, Da-Eun Yoon, Younbyoung Chae

**Affiliations:** 1 School of Artificial Intelligence in Traditional Chinese Medicine, Shanghai University of Traditional Chinese Medicine, Shanghai, China; 2 Department of Meridian and Acupoints, College of Korean Medicine, Kyung Hee University, Seoul, Republic of Korea

**Keywords:** Acupuncture dose, Closed-loop control, *Deqi*, Kinematics, Manipulation

## Abstract

The clinical effects of acupuncture depend heavily on its physical parameters, particularly treatment dose and needling technique. Yet these parameters are still poorly standardized in both research and clinical settings. This reproducibility, hinders interpretation of findings, and complicates practitioner training. In this article we synthesize recent work together to propose a closed-loop framework for precision acupuncture, organized around three linked stages. The input stage defines dose mainly in terms of stimulation intensity and translates traditional diagnostic reasoning into a small set of measurable manipulation parameters. The process stage concerns how that stimulation is physically delivered, how it can be quantified through kinematic metrics, and how it can be taught through sensorimotor training. The control stage uses *deqi* as a real-time feedback signal, while candidate pretreatment biomarkers are considered as feedforward modifiers for initial dose selection. Taken together, these developments support a more quantitative and technology-assisted approach that preserve traditional clinical reasoning while making acupuncture delivery measurable and reproducible.

## Introduction

Evidence suggests that acupuncture may exhibit dose–response relationships. Among the dose components examined, variation in stimulation intensity has shown more consistent associations with clinical outcomes than variation in needle number or treatment frequency, although the available evidence remains limited and does not permit formal weighting of these components [1]. Tonification and sedation can be read as two ends of a stimulation-intensity spectrum, and neuroimaging work indicates that higher doses engage sensorimotor brain regions more robustly [2, 3]. On this view *deqi* is not the dose itself but a rapid, clinically accessible sign that stimulation has been adequate, which enables individualized dose adjustment during treatment. It remains unclear how specific stimulation parameters map onto physiological responses and clinical effects.

Learning to needle well demands fine, nuanced motor skill, yet acupuncture has traditionally been taught and assessed by largely subjective means. The parameters that matter—manipulation type, duration, needling velocity, and applied force—are all tied to the stimulation dose that is actually delivered. When practitioners are studied quantitatively, their movements vary considerably from one person to the next but stay remarkably consistent within a given individual, complicating standardization [4]. Several tools have been developed to address this: high-fidelity phantom acupoints that reproduce the feel of needle grasp, sensor systems that give real-time visual feedback on motion, and three-dimensional motion tracking that captures how experts move [5].

Here we bring recent evidence together into a closed-loop framework for precision acupuncture, organized into three phases. The input phase treats stimulation intensity as the main determinant of dose and recasts traditional diagnostic categories as measurable kinematic parameters. The process phase addresses the physical delivery of stimulation by validating biomechanical interfaces and quantifying manipulation through motion analysis and simulation-based training. The control phase uses *deqi* as a real-time response sensor and considers emerging biological biomarkers as possible predictive indicators for dose optimization. Our aim is to outline a path toward a more standardized and quantifiable practice, while preserving the value of clinical experience.

## Input phase: defining dose and strategic parameters

### Operational definition: stimulation intensity as the primary input

Acupuncture dose can be broken down into three components: the number of needles, the frequency and cumulative count of sessions, and stimulation intensity [1]. Of these, stimulation intensity appears to be a major, but not exclusive, determinant of the dose–response relationship. It captures several aspects of technique at once—the amplitude and frequency of manipulation, the retention time, and the insertion depth—and can be readily adjusted to modulate the delivered dose [1]. For that reason, we treat stimulation intensity as the primary input variable in the proposed closed-loop model.

In our earlier systematic analysis, needle number and treatment frequency did affect outcomes, but their individual contributions were smaller and less consistent than that of stimulation intensity, and they tended to interact with intensity rather than act independently [1]. A formal weighting of each factor is not yet possible from the available data, because most trials change several parameters at the same time. We therefore regard stimulation intensity as the primary, but not sole, determinant of dose. Quantifying the relative contribution of each component remains an open question for future dose-finding studies.

### Strategic input: translating traditional diagnosis into quantitative metrics

Tonification and sedation correspond to deficiency and excess patterns, respectively. They can be understood as different levels of stimulation intensity: tonification uses weaker stimulation for deficiency patterns, sedation uses stronger stimulation for excess patterns, and the choice is guided by the patient's overall condition, constitution, and pulse findings [1, 2, 6]. In practice, then, clinicians titrate intensity to the individual, using *deqi* and the patient's general response as their guides.

Quantitative studies support this intensity-based reading. Compared with tonification, sedation-oriented manipulations tend to involve larger rotation amplitudes, higher movement frequencies, and longer continuous periods of manipulation [4, 7]. Quantitatively, sedation is generally delivered with rotation amplitudes above about 90° and manipulation frequencies over roughly 2 Hz, sustained for longer stretches, whereas tonification uses gentler input—smaller rotation amplitudes (below about 90°), lower frequencies (on the order of 1 Hz or less), shorter continuous manipulation, and a shallower, slower lifting–thrusting motion [6, 7]. However, universally validated cutoffs for manipulation amplitude or frequency have not yet been established.

These parameters must be implemented with biomechanical precision through standardized manipulation techniques. By “standardized manipulation techniques” we mean reproducible protocols that specify the manipulation type (lifting–thrusting, rotation, or a combination), the target ranges for amplitude and frequency, the insertion depth and retention time, and the total duration of stimulation—rather than leaving these to undocumented personal habit. Such protocols build on existing textbook and national-standard definitions of tonification and sedation, and they become genuinely auditable only when the delivered motion is measured against them using the kinematic tools described in next section.

## Process phase: quantification and educational scaling

### Biomechanical interface: phantom acupoints

Before the delivery of stimulation can be quantified, the physical interface where needling happens has to be validated. To give trainees a safe, repeatable, and realistic medium for practice, researchers have developed and validated phantom acupoints [8]. Usually made from agarose gel, these can be tuned by changing the gel concentration to reproduce the mechanical forces and the characteristic “needle grasp” of different human acupoints. They have proved a useful teaching tool: phantom-based programs measurably improve the basic technical skills of novice students [9], and they offer an inexpensive, portable, and ethically uncomplicated way to rehearse depth control and timing.

### Kinematic metrics and digital signatures

With validated phantoms, motion- and force-sensing technologies can record displacement, rotation, frequency, and force continuously during manipulation. As noted, these analyses show marked differences between practitioners but strong consistency within each one, so that every practitioner exhibits a stable motion “signature” [4]. This pattern of high between-practitioner variability and strong within-practitioner reproducibility helps explain why acupuncture has been regarded as an “art” and identifies the need for tools to quantity and standardize its practice.

Three-dimensional motion tracking breaks complex hand movements down into sinusoidal waveforms defined by amplitude and frequency, producing digital signatures of expert manipulation [7]. Data of this kind support more empirical training and the development of intelligent systems. These include a robot-assisted devices that reproduce expert manipulations while monitoring motion and force, as well as mixed-reality platforms combining virtual avatars with haptic feedback.

Machine-learning methods take this further, turning motion and tactile datasets into automated assessment. Vision-based deep learning and three-dimensional hand-pose estimation can classify manipulation type and estimate amplitude and frequency in real time. These methods provide immediate feedback on consistency and on deviations from an expert reference. Together, these methods transform the process phase from a “black box” into a measurable and controllable component of dose delivery.

Because these vision- and force-based systems already capture amplitude, frequency, depth, and duration during needling, the same data stream can be used to generate a structured, time-stamped record of each treatment automatically. Practitioners could then document intensity-related parameters as an auditable clinical record without the burden of manual note-taking, and the resulting standardized data would be exactly what is needed to connect specific doses to outcomes across different clinics.

### Scaling standardization: sensorimotor training for acupuncture manipulation

**Fig. 1:**
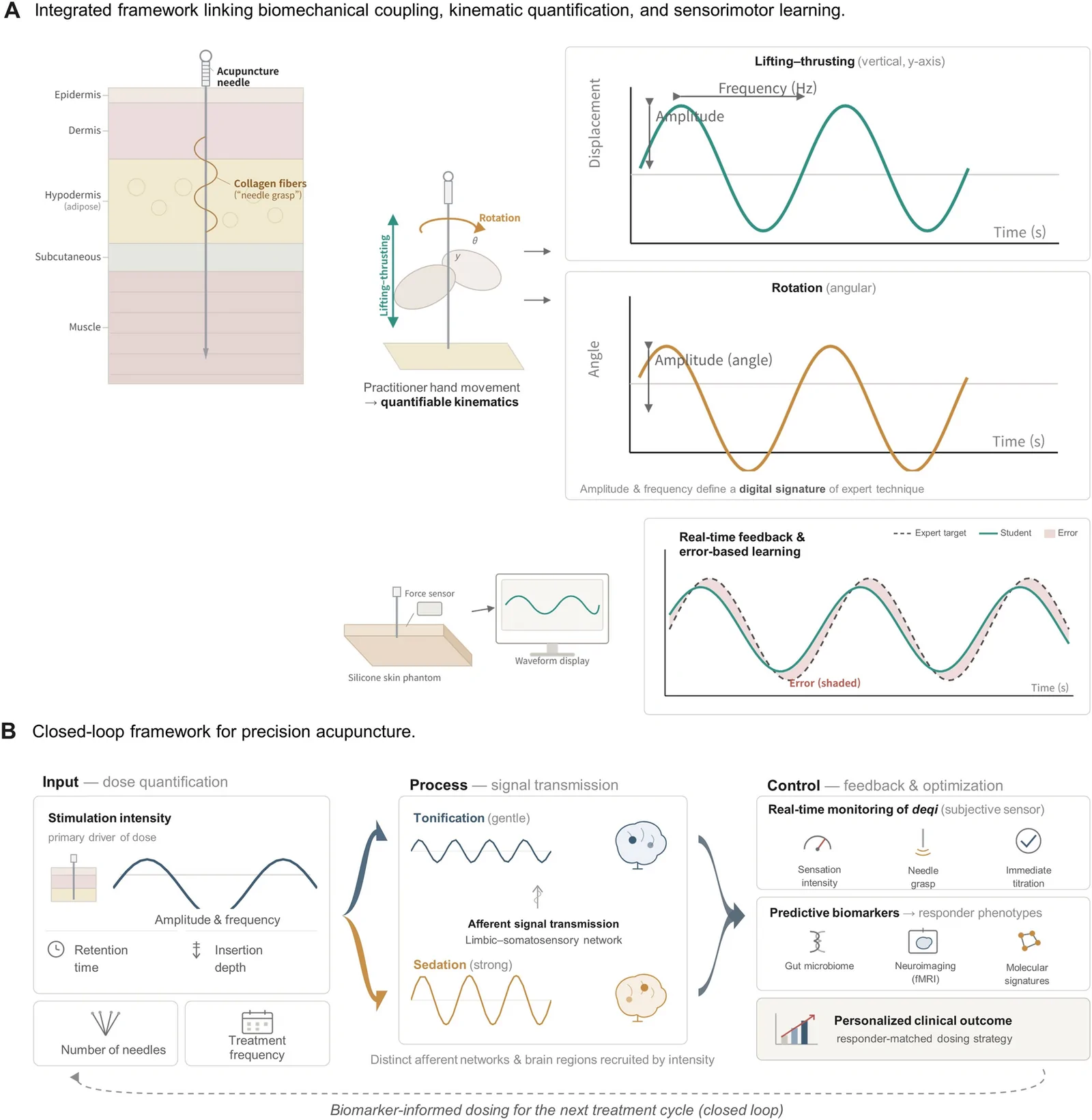
A proposed closed-loop framework for quantifiable acupuncture delivery and feedback. **A** Integrated framework for biomechanical coupling, kinematic quantification, and sensorimotor learning in acupuncture. During manipulation, collagen winding around the needle shaft contributes to needle grasp and local biomechanical coupling, although its specific relationship to the *deqi* sensation remains to be established. Complex practitioner hand movements are decomposed into quantifiable kinematic metrics: lifting–thrusting (vertical *y*-axis) and rotational (angular) components are represented as sinusoidal waveforms defined by amplitude (displacement or angle) and frequency (Hz), establishing a digital signature of expert technique. A phantom system fitted with force sensors provides immediate visual feedback, with the student's performance (real-time trajectory, teal line) superimposed on an expert template (target waveform, dashed line); the resulting error (shaded red area) is highlighted to support error-based learning. **B** Closed-loop framework for precision acupuncture. (1) Input phase (dose quantification): stimulation intensity (amplitude and frequency) is defined as the primary driver of dose, together with physical parameters such as insertion depth and retention time. (2) Process phase (signal transmission): these kinematic inputs modulate neural engagement, with “tonification” (gentle stimulation) and “sedation” (strong stimulation) potentially recruiting different afferent networks and brain regions. (3) Control phase (feedback and optimization): the system operates as a closed loop through two layers of control—with-session monitoring of *deqi* (subjective sensor) enables immediate titration of manipulation, while candidate biomarkers (e.g., gut microbiome profiles and neuroimaging features) may help stratify patients into responder phenotypes. These candidate markers may eventually inform the initial dosing strategy for subsequent treatment cycles, supporting personalized clinical outcomes

The Acupuncture Manipulation Education System (AMES) is a concrete example of sensorimotor training built on these metrics [5]. AMES tracks needle movement and displays the learner's trajectory as it is generated, superimposed on an expert waveform template, so that error is displayed visually and can be corrected quickly. Novices trained with AMES improve their motion fidelity and reduce error on phantom acupoints. Whether these gains transfer to human needling has not yet been established. This provides a scalable approach to training practitioners to deliver precise and reproducible stimulation (Fig. 1A).

## Control phase: response monitoring and predictive indicators

### Real-time sensor: Deqi as a response indicator

Patients differ in how they respond, which is why the field distinguishes “high responders” from “low responders” and why individualized titration matters. *deqi*—the heaviness, soreness, or distension patients report during treatment—serves as the main immediate indicator of the patient’s response to stimulation. It is best regarded as a response signal, rather than a dose component, that guides real-time adjustment of amplitude, frequency, and duration.

Clinical and neurophysiological studies tie appropriate *deqi* to a greater chance of symptom improvement, especially in pain and in autonomic or cardiovascular conditions. Its presence, quality, and time course track with manipulation parameters and with activation of the limbic–paralimbic–neocortical networks involved in interoception and affective processing. In some conditions a shorter *deqi* latency has been linked to faster improvement, which suggests that its timing may index how sensitive a given patient is to stimulation.

### Emerging biological biomarkers as feedforward predictors

Because *deqi* is subjective and condition-specific, there is growing interest in objective biomarkers that might predict responsiveness before treatment even begins. Four classes have attracted most attention: gut microbiome profiles, neuroimaging-based markers, inflammatory cytokine levels, and circulating microRNA panels. Early reports link baseline microbial taxa to response in postpartum depression, pre-treatment MRI features to outcomes in migraine and mild traumatic brain injury, cytokine levels to pain responsiveness, and microRNA signatures to chronic pain severity [10].

Whether these are worth pursuing depends on several practical criteria. A candidate marker should be reproducible across laboratories, biologically plausible in relation to known acupuncture mechanisms, and measurable using reasonably accessible samples. Most importantly, it should prospectively predict response rather than merely correlate with it. Unlike *deqi*, pretreatment biomarkers do not constitute feedback in the engineering sense. Within the proposed framework, they are better regarded as feedforward modifiers that may help define an initial dose range, whereas *deqi* provides within-session feedback for ongoing dose adjustment.

These biomarkers remain exploratory, and their clinical translation faces both scientific and practical barriers. Most studies are small and single-center, and no trial has yet shown that biomarker-guided dosing beats experienced clinical judgment. Just as relevant to everyday practice, multi-omics assays and advanced neuroimaging are still costly, depend on specialized equipment and analytic expertise, and have turnaround times poorly suited to point-of-care decisions. In most real-world clinics, particularly community and primary care settings, they are not yet feasible or cost-effective. For the foreseeable future, biomarkers are therefore best treated as research tools, and eventually as a way to stratify selected patients, not as routine tests. Within the closed loop, they are best seen as complementary to *deqi*: they may one day inform initial intensity ranges and patient stratification, while *deqi* continues to provide the primary intra-session feedback for real-time titration (Fig. 1B).

## Conclusions

The evidence reviewed here supports a more measurable and standardized approach to acupuncture, structured as a closed loop from a quantified input, through a physically validated and measurable process, to *deqi-*centered feedback control. Phantom models, kinematic monitoring, and sensor-based feedback already make parts of this loop feasible, providing a practical approach to more objective and transferable acupuncture training.

However, a fully closed-loop clinical system has not yet been achieved. The kinematic tools have mostly been validated on phantoms, not on patients, the predictive biomarkers are still at an early stage, and it has not yet been shown in controlled trials that quantitative, feedback-guided dosing produces better outcomes than the judgment of an experienced practitioner. This framework should be viewed as a research agenda that complement clinical expertise with measurement, instead of as an established standard of care.

## Data Availability

No datasets were generated or analysed during the current study.

## References

[1] Yoon DE, Lee IS, Chae Y. Identifying dose components of manual acupuncture to determine the dose-response relationship of acupuncture treatment: a systematic review. Am J Chin Med. 2022;50(3):653–71. 35300569 10.1142/S0192415X22500264

[2] Yoon DE, Lee IS, Chae Y. Comparison of the acupuncture manipulation properties of traditional East Asian medicine and Western medical acupuncture. Integr Med Res. 2022;11(4):100893. 36353444 10.1016/j.imr.2022.100893 PMC9637804

[3] Yoon DE, Lee S, Kim J, Kim K, Park HJ, Napadow V, et al. Graded brain fMRI response to somatic and visual acupuncture stimulation. Cereb Cortex. 2023;33(23):11269–78. 37804240 10.1093/cercor/bhad364

[4] Seo Y, Lee IS, Jung WM, Ryu HS, Lim J, Ryu YH, et al. Motion patterns in acupuncture needle manipulation. Acupunct Med. 2014;32(5):394–9. 24938530 10.1136/acupmed-2014-010585

[5] Lee YS, Jung WM, Lee IS, Lee H, Park HJ, Chae Y. Visualizing motion patterns in acupuncture manipulation. J Vis Exp 2016(113). 10.3791/54213 27501193

[6] Yoon DE, Jang W, Ryu Y, Lee IS, Chae Y. Characterizing the stimulation intensity in acupuncture manipulation techniques for tonification and sedation therapy. Korean J Acupunct. 2022;39(3):91–6.

[7] Tang WC, Yang HY, Liu TY, Gao M, Xu G. Motion video-based quantitative analysis of the “lifting-thrusting” method: a comparison between teachers and students of acupuncture. Acupunct Med. 2018;36(1):21–8. 28844062 10.1136/acupmed-2016-011348 PMC5865511

[8] Lee IS, Lee T, Shin WC, Wallraven C, Lee H, Park HJ, et al. Haptic simulation for acupuncture needle manipulation. J Altern Complement Med. 2014;20(8):654–60. 25020028 10.1089/acm.2013.0475

[9] Lee IS, Lee YS, Park HJ, Lee H, Chae Y. Evaluation of phantom-based education system for acupuncture manipulation. PLoS ONE. 2015;10(2):e0117992. 25689598 10.1371/journal.pone.0117992 PMC4331364

[10] Zhou Y-M, Yuan J-J, Xu Y-Q, Gou Y-H, Zhu YY, Chen C, et al. Fecal microbiota as a predictor of acupuncture responses in patients with postpartum depressive disorder. Front Cell Infect Microbiol. 2023;13:1228940. 38053532 10.3389/fcimb.2023.1228940 PMC10694210

